# Effect of ambient lighting on frequency dependence in transcranial electrical stimulation-induced phosphenes

**DOI:** 10.1038/s41598-022-11755-y

**Published:** 2022-05-11

**Authors:** Ian Evans, Stephen Palmisano, Rodney J. Croft

**Affiliations:** 1 School of Psychology, University of Wollongong, Wollongong, Australia; 2 Illawarra Health and Medical Research Institute, University of Wollongong, Wollongong, NSW 2522 Australia; 3 Australian Centre for Electromagnetic Bioeffects Research, Wollongong, Australia; 4 Centre for Population Health Research On Electromagnetic Energy, Monash University, Melbourne, Australia

**Keywords:** Neuroscience, Psychology

## Abstract

Inconsistencies have been found in the relationship between ambient lighting conditions and frequency-dependence in transcranial electric stimulation (tES) induced phosphenes. Using a within-subjects design across lighting condition (dark, mesopic [dim], photopic [bright]) and tES stimulation frequency (10, 13, 16, 18, 20 Hz), this study determined phosphene detection thresholds in 24 subjects receiving tES using an FPz-Cz montage. Minima phosphene thresholds were found at 16 Hz in mesopic, 10 Hz in dark and 20 Hz in photopic lighting conditions, with these thresholds being substantially lower for mesopic than both dark (60% reduction) and photopic (56% reduction), conditions. Further, whereas the phosphene threshold-stimulation frequency relation increased with frequency in the dark and decreased with frequency in the photopic conditions, in the mesopic condition it followed the dark condition relation from 10 to 16 Hz, and photopic condition relation from 16 to 20 Hz. The results clearly demonstrate that ambient lighting is an important factor in the detection of tES-induced phosphenes, and that mesopic conditions are most suitable for obtaining overall phosphene thresholds.

## Introduction

Many aspects of neural processing rely on frequency-specific oscillations in the cortex^1^. As a result, the possibility of exploring and/or manipulating these frequency-based neural functions using a non-invasive technique like transcranial electric current stimulation (tES) has proven popular. Applying electric current to the brain using tES has been demonstrated to be successful in modulating cognitive, sensory, and motor functions in a frequency-dependent manner across the surface of the cortex (for reviews see^2,3^). Although tES can modulate cortical activity, it can also induce phosphenes (i.e., perceptions of light that are not the product of external visual stimuli^4–6^). These phosphenes are generally considered to be a product of electrical stimulation of the retina^6–12^.

It is important to understand both the biological mechanisms responsible for inducing phosphenes, and any environmental factors that influence their appearance, as these can confound tES studies, interventions and interpretations^13^. For example, the threshold for inducing phosphenes is currently used by the International Commission on Non-Ionizing Radiation Protection (ICNIRP) for deriving exposure restrictions^14^, and that information can only be obtained if the effect of stimulation frequency and ambient lighting conditions are also known. Without that knowledge, experimentally-derived threshold estimates may merely represent the lowest stimulation levels required to induce phosphenes in a particular *insensitive* scenario, which would limit the ability of exposure restrictions based on them to protect against phosphenes in other situations. Indeed, recent research suggests that our understanding of phosphenes may be particularly limited in terms of their relation with stimulation frequency and ambient lighting conditions.

It has commonly been held that thresholds for phosphenes induced by transcranial alternating current stimulation (tACS) are lowest when stimulation is applied at 20 Hz in photopic (i.e., intense) lighting conditions and at 10 Hz in complete darkness^15^; sensitivities that closely match the dominant frequencies of the visual cortex oscillations observed under these respective lighting conditions (e.g.^16,17^). These findings have been taken as evidence that tES, tuned to the dominant cortical oscillation frequency, can be used to maximally modify cortical activity at similar rhythms.

However, recent research suggests that under mesopic (dim) lighting conditions, tES phosphenes are induced with considerably less current using 16 than 20 Hz stimulations^18,19^, which suggests that 20 Hz does not provide the lowest stimulation level required to induce phosphenes. This raises concerns about the relative veracity of these new reports. The only research available for comparison explicitly testing phosphene threshold levels in mesopic conditions is the Schwarz^20^ study. Schwarz^20^ reported lower phosphene detection thresholds for 20 Hz stimulation in both photopic (i.e., 8–9550 cd per square meter; cd/m^2^) and mesopic (i.e., 2.4 cd/m^2^) conditions. However, those findings were based on only a single subject and used poorly controlled lighting conditions; e.g. the 2.4 cd/m^2^ condition was produced by having the subject look at “her own shadow on the wall”, and the 9550 cd/m^2^ condition was produced by having the subject look at “a white cloud in the sky”. This makes it difficult to draw conclusions from such a comparison. In contrast to Schwarz’s^20^ research, more recent studies^18,19^ used considerably larger samples (either 24 or 22 participants in each of these studies) with tightly controlled lighting (consistent 0.6 cd/m^2^ lighting across the entire field of view). Furthermore, the consistency of the initial^18^ and replication^19^ study suggests that their results are indeed reliable. This conclusion, however, would appear (at face value) to contradict the view that the greatest sensitivity to tES occurs at the stimulation frequency that matches the dominant cortical oscillation frequency. That is, whereas the dominant cortical oscillation frequencies for photopic and dark conditions are approximately 20 and 10 Hz respectively, and the lowest current required to induce phosphenes is at 20 and 10 Hz respectively, there is no corresponding dominant frequency for mesopic conditions.

One potential explanation for lower thresholds at 16 Hz stimulation is that this represents an overlap point between the threshold-stimulation frequency relations for photopic and dark conditions. That is, as a dim lighting condition represents a degree of photic energy that is greater than in the dark, but less than in a photopic scenario, it may be relevant to the threshold-stimulation frequency of both the dark and photopic conditions. To test this possibility, threshold-stimulation frequency relations need to be assessed under each of dark, mesopic and photopic conditions.

Differences in frequency dependence found in tES-induced phosphenes across lighting conditions may be explained by differences in temporal contrast sensitivity functions (i.e., the visual system’s sensitivity to changes in luminance over time^21,22^). Temporal contrast sensitivity is typically measured using a homogenous visual stimulus that changes sinusoidally in luminance (from a minimum to a maximum value) as a function of time. While this stimulus should be perceived to flicker with larger levels of luminance contrast, it will become progressively more difficult to see this flicker as this luminance contrast decreases. However, the threshold level of luminance contrast at which this flicker is just noticeable also depends critically on the temporal frequency of the stimulus. Research has shown that rod and cone photoreceptors each have their own temporal contrast sensitivity functions, which may relate to differences in tES-related frequency dependence found in different lighting conditions.

Rod photoreceptors are more sensitive to stimulation (and thus more likely to be activated) in darker conditions, where there is insufficient input to activate cones^23^. In dark and mesopic conditions, temporal contrast sensitivities are largely driven by inputs from rod photoreceptors^24^ particularly when exposed to stimuli flickering at 5–15 Hz while showing little to no activation at 19–23 Hz^25,26^. On the other hand, cone photoreceptors are more sensitive to stimulation in brighter conditions, where rod cells are saturated and do not contribute significantly to visual perception^27^. Temporal contrast sensitivity in these photopic conditions trends towards higher frequencies, with greatest sensitivity found at around 15–25 Hz and no ability to discern stimuli flickering at or above approximately 80 Hz^25,26,28^. If the perception of tES-induced phosphenes is similar or functionally equivalent to external flickering visual stimuli^6^, it would follow that darker conditions would result in greater sensitivity to low-frequency stimulation, and brighter conditions would result in greater sensitivity to higher frequencies.

The present study was designed to determine the following. First, by testing the threshold-frequency stimulation relation in each of dark, mesopic and photopic conditions, it examined whether the overall phosphene detection threshold occurs at 16 Hz stimulation, rather than 20 Hz. Second, it extended the results of previous studies^18,19^, which found lower thresholds at 16 Hz stimulation in mesopic conditions (and which increased for higher and lower frequencies), compared to dark and photopic lighting conditions. Third, it determined whether the phosphene threshold-stimulation frequency relation under mesopic conditions could be explained by the overlap of that relation across dark and photopic conditions.

## Method

Twenty-four healthy participants (even gender split, age range 20–40 years, *M* = 25.2 years, *SD* = 5.4) completed this study after passing a modified safety screening checklist^29^. Participants were excluded if they reported any form of neural injury or illness, metal implants in the head or medical implant elsewhere in the body, or non-corrected visual impairment. No participants reported using contact lenses, while three participants typically wore glasses but removed them during the testing phase to ensure the frames did not alter the periphery of their field of view. After being informed about the experimental procedure as well as potential adverse effects of tES, subjects gave written and informed consent prior to any participation. This research was conducted in accordance with the guidelines of the Declaration of Helsinki, and approved by the Human Research Ethics Committee of the University of Wollongong (approval #HE2017/454).

**Figure 1:**
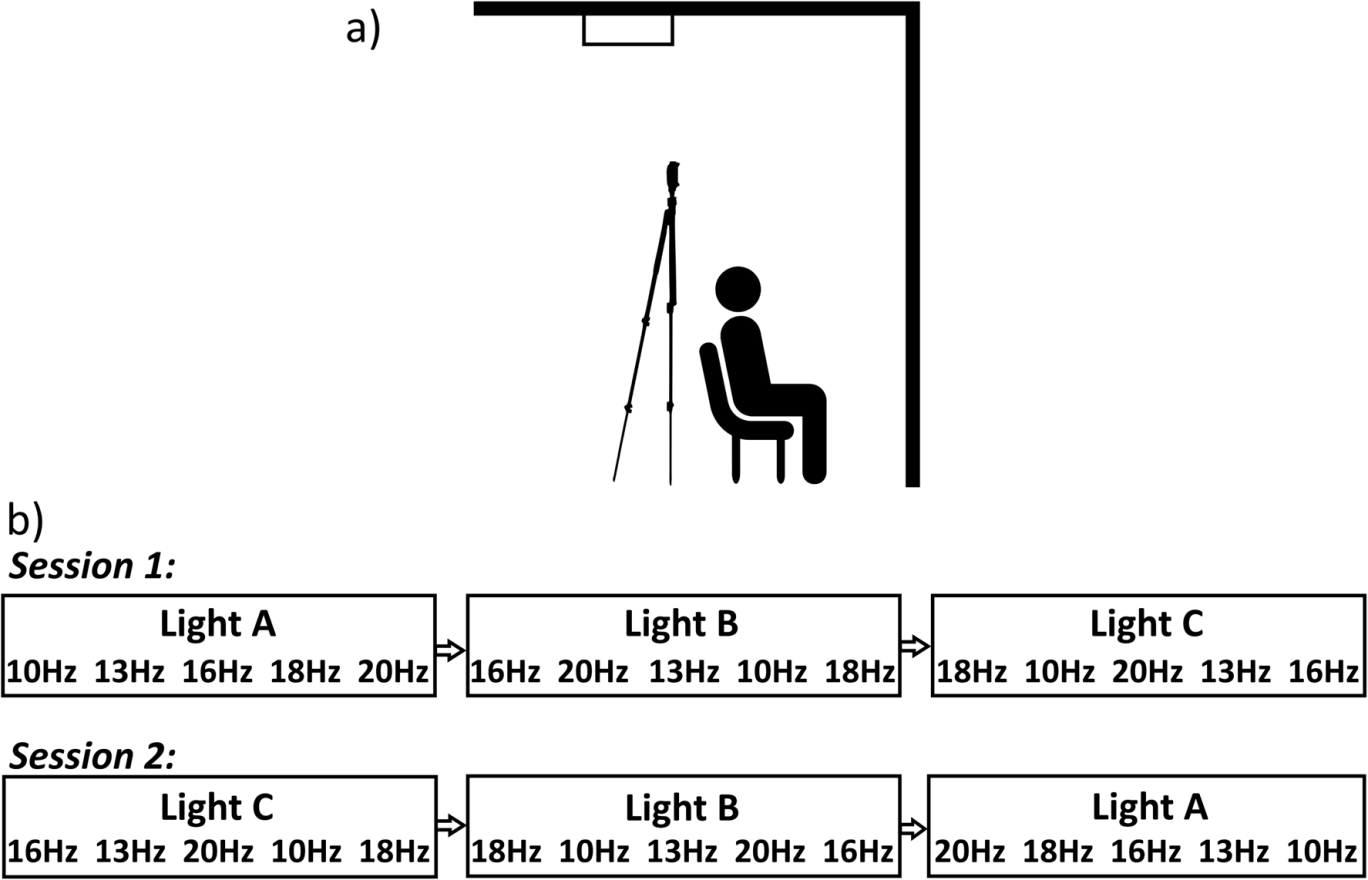
(**a**) Positioning of lighting and test subject. The test subject was seated so that the front wall filled their entire field of view (no parts of either side wall were visible). The light stand was positioned so that no shadows were visible in the subject’s field of view. The photopic lighting condition was achieved by activating the fixed fluorescent ceiling light with no light stand used, while all other lighting conditions (the mesopic condition and the brief periods requiring light in the dark condition) were achieved using the light stand only. (**b**) Example sequence of the block design across both sessions. For the first session, each lighting condition block was presented in a random order, as were the frequencies within it. In order to account for potential within-subject order effects due to possible light adaptation, in the second session the order of the lighting blocks was reversed and the frequencies within those blocks were reversed.

Phosphene thresholds were obtained as a function of stimulation frequency (“Frequency”: 10, 13, 16, 18, and 20 Hz) and lighting condition (“Lighting Condition”: dark, mesopic, photopic), using a repeated measures design. Testing was conducted over two, 70-min sessions (on separate days) at similar times of day and usually within one week of each other. The order of these sessions, the order of the tES montages within these sessions, and which electrode was the cathode or anode within each montage, were counterbalanced across sessions for all participants (see Fig. 1b). The choice of which electrode was cathode/anode alternated across sessions for each subject. The order of the lighting conditions was randomised for each participant using a Latin square system, as was the order of stimulation frequency within each lighting condition.

tES was administered using a Magstim NeuroConn DC Stimulator Plus MOP15-EN-01 (Magstim, Carmarthenshire, UK) which applied sinusoidal DC with no ramp-up, meaning that the amplitude of the stimulation varied as a sine wave from zero to the current set by the stimulator then back to zero. As such, the polarity of the electrodes did not alternate. Current was delivered to the scalp through conductive-rubber electrodes (dimensions: 3 × 4 cm) placed on sponges saturated with a saline solution mixed with a hypoallergenic amphoteric surfactant and held in place at FPz and Cz with rubber straps. This electrode montage was chosen as it is effective at stimulating the retinas^30^ while also ensuring the participants’ entire field of view was not occluded by any of the apparatus. Previous tES studies under mesopic lighting conditions^18,19^ all consistently found greatest sensitivity at 16 Hz using a wide range of montages (FPz-Cz, Oz-Cz, FPz-Oz, T3-T4), suggesting that the choice of montage does not appreciably affect the frequency-dependent nature of tES-induced phosphenes providing that the retina is adequately stimulated.

The photopic lighting condition was generated by using typical ceiling-mounted fluorescent lights, positioned outside the participants’ direct line of sight. Under these lighting conditions, luminance at the eye was measured at 77.1 ± 0.05 cd/m^2^ using a J6523 Tektronix luminance probe (Tektronix, London, Canada). This lighting level was chosen due to its applicability to everyday experience, as it represents the luminance typically found in office environments. The mesopic lighting condition was created using the Neewer T120 dimmable LED panel illuminating the areas in front of the participant (see Fig. 1a), resulting in luminance at the eye measured at 0.6 ± 0.05 cd/m^2^. This lighting level was chosen in order to match that of previous studies^18,19^ and thus to enable replication of their results. No light entered the testing room during the dark condition, resulting in zero cd/m^2^. In order to prevent dark adaptation effects during the trials in the dark condition, lighting was set to 1.1 cd/m^2^ while the stimulator was not active; lighting was turned off 2 s prior to stimulation onset and turned on immediately after the stimulation had ended.

Participants were seated on a chair facing a 1.8 m wide by 2.62 m high white wall, in a position that ensured that the wall in front of them filled their entire field of view, and lighting was arranged so that no shadows were visible to the participant (see Fig. 1a). When participants were comfortable and the electrodes put in position, they were informed about phosphenes (their nature and what they might perceive) whilst their skin and hair were saturated from the saline in the sponges. Once impedance between the electrodes was at 15 kΩ or below (as indicated by the stimulator), lighting was set to 2 ± 0.05 cd/m^2^ and participants were familiarized with the appearance of phosphenes using 10 s of sinusoidal tES at 1000 μA, firstly at 11 Hz and then at 22 Hz, to demonstrate both the visual appearance of phosphenes, and how their appearance changes by simply varying the stimulation frequency. These frequencies were chosen to avoid using the same stimulation frequencies as in the experiment itself.

Once participants were familiarized with phosphenes, their phosphene detection thresholds were determined at each stimulation frequency and lighting condition. They were informed when each stimulation began and when it ceased, but they were not informed of the frequency or current intensity of the stimulation. Stimulations lasted for 5 s, and participants were instructed to keep their eyes open throughout the entire stimulation. Throughout the experiment participants were asked how bright the phosphenes appeared to be compared to the background lighting, and where the phosphenes appeared in their field of view. Many participants also spontaneously volunteered information concerning their experience during the interval between trials. To detect false positive responses at lower current intensity levels, six sham stimulations (one in each lighting condition for each session) were given at a frequency determined in advance using a MATLAB-based random number generator. In these sham trials, the participant was given all the audible signs of a stimulation (the usual button presses on the stimulator as well as verbal indications that the stimulation had started and finished) without actually generating an electric current. None of the participants reported seeing phosphenes during any of the sham trials.

Thresholds for phosphene induction (in μA) were determined for each frequency by varying the current intensity using a QUEST-based Bayesian adaptive staircasing procedure^31^ in MATLAB’s PsychToolbox^32^. The tES, which started at 700 μA, was bound between 25 and 1500 μA. The step-size between possible stimulation levels was 25 μA. Based on the Rapid Estimation of Phosphene Threshold system validated by Mazzi et al.^33^, this adaptive threshold measurement method determined the lowest current intensity that was significantly more likely than chance to evoke phosphene perceptions. Each of the two sessions provided a threshold for each lighting and frequency condition, and for each combination of lighting condition and stimulation frequency, the average threshold across both sessions was taken as the final threshold.

### Statistical analysis

As significant levels of skewness, kurtosis or heterogeneity of variance were not found, parametric analyses were conducted. Huynh–Feldt adjustments were used to account for violations to sphericity (Frequency; Frequency by Lighting Condition), with the adjusted degrees of freedom shown.

To assess threshold reliability across the two testing sessions, for each of the Lighting Condition (photopic, mesopic, dark)/Frequency (10, 13, 16, 18, 20 Hz) combinations, Pearson’s *r* was determined. To determine if order effects were distorting the results, thresholds were arranged in chronological order and repeated measures ANOVA were used where threshold was the dependent variable and testing order within each lighting condition (separately for each session) and across each entire session were the independent variables.

To describe the relations between phosphene thresholds, lighting conditions and stimulation frequency, a repeated measures ANOVA was used where threshold was the dependent variable, and Lighting Condition and Frequency the independent variables. Where significant, data were further explored using repeated measures *t* tests with Bonferroni comparison-wise adjustments (Lighting Condition: each level was compared to each other level; Frequency: each level was compared to each other level; Interaction: for each frequency, each level of Lighting Condition was compared to each other level). Adjusted p-values are shown.

To determine the lowest absolute phosphene thresholds across the lighting conditions, a repeated measures ANOVA was used where Lighting Condition was the independent variable, and the dependent variable was the lowest algebraic threshold across the frequencies, for each lighting condition separately. Where the main effect was significant, *t* tests with Bonferroni comparison-wise adjustments (Lighting Condition: each level was compared to each other level) were conducted. Adjusted p-values are shown.

To determine whether the phosphene threshold-stimulation frequency relation in the mesopic condition could be adequately explained by the summation of the relations in the dark and photopic conditions, regression equations were calculated as follows. To provide an estimate of the phosphene threshold-stimulation frequency relation for each of the lighting conditions separately, regression analyses were conducted for each lighting condition separately where threshold was the dependent variable (normalized across all tested frequencies, within each subject and lighting condition separately), and Frequency the independent variable. Corrected Akaike’s Information Criteria (AICc^34^) was used to determine whether a linear or quadratic fit was the best model for each lighting condition. Data for each participant was used for all frequencies and lighting conditions, resulting in 72 data points per frequency.

## Results

**Figure 2:**
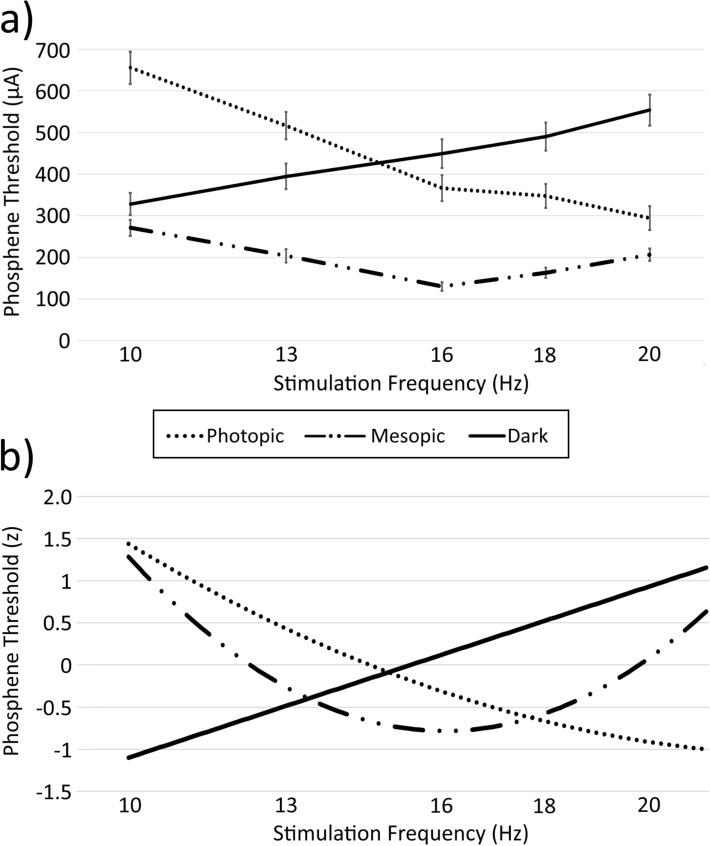
(**a**) Phosphene thresholds and standard errors for each ambient lighting condition at each frequency tested (10, 13, 16, 18 and 20 Hz). (**b**) Regression-based estimates of normalised phosphene thresholds, as a function of stimulation frequency, for each ambient lighting condition.

Thresholds at each Frequency/Lighting Condition combination can be seen in Fig. 2a and in Supplementary Table 1. The interpolated regression functions relating threshold to Frequency, for each lighting condition separately, can be seen in Fig. 2b. Corresponding means and standard errors are given in Table 1.

**Table 1:** Means (and standard errors) of the phosphene thresholds for each frequency and lighting condition tested. n = 24.

Frequency (Hz)	Photopic	Mesopic	Dark
10	655.2 (39.2)	270.8 (19.2)	328.1 (26.9)
13	516.1 (32.7)	203.6 (16.0)	394.3 (30.6)
16	366.1 (31.0)	130.2 (10.2)	449.0 (34.5)
18	347.4 (28.3)	163.0 (12.5)	490.1 (33.8)
20	294.3 (28.6)	206.3 (14.9)	553.6 (37.5)

Phosphene thresholds were highly reliable across the two testing sessions, with Pearson’s r coefficient values ranging from 0.91 to 0.99 (all *p* < 0.001) across the 15 Frequency/Lighting Condition combinations. No signs of learning or fatigue effects were found within either session (*p* between 0.707–0.883) or within each lighting condition (*p* between 0.472–0.940). Mean thresholds for all (30) testing points ranged between 313.5–407.3 µA, with standard deviations between 166.3–246.4 µA.

Phosphene thresholds were affected by lighting condition (main effect: *F*(1.96,45.16) = 59.15, *p* < 0.001, $\eta_{p}^{2}$ = 0.720), with post hoc analyses showing that this was due to lower thresholds in the dim lighting condition relative to both the photopic (*F*(1, 23) = 116.4, *p* < 0.001, $\eta_{p}^{2}$ = 0.835) and dark (*F*(1, 23) = 93.93, *p* < 0.001, $\eta_{p}^{2}$ = 0.803) conditions; no difference was observed between the photopic and dark conditions (*p* > 0.999). Phosphene thresholds (for the combined lighting conditions) were also affected by Frequency (main effect: *F*(1.69,38.91) = 26.46, *p* < 0.001, $\eta_{p}^{2}$ = 0.535). The frequency with the lowest threshold (16 Hz) was lower than each other frequency (all *p* < 0.049), and the frequency with the highest threshold (10 Hz) was higher than each other frequency (*p* < 0.004). Of the remaining comparisons, thresholds for 13 Hz were higher than 18 Hz (*F*(1, 23) = 10.34, *p* = 0.038, $\eta_{p}^{2}$ = 0.310) but did not differ from 20 Hz (*p* = 0.795), and 18 Hz was lower than 20 Hz (*F*(1, 23) = 17.75, *p* = 0.003, $\eta_{p}^{2}$ = 0.436).

The interaction between Frequency and Lighting Condition was also significant *F*(4.63,106.50) = 117.60, *p* < 0.001). Follow-up analyses for the significant interaction demonstrated the following: *10 Hz Stimulation.* Thresholds were lower for both the mesopic (*t*(23) = 13.77, *p* < 0.001, Cohen’s *d* = 2.81) and dark (*t*(23) = 8.85, *p* < 0.001, *d* = 1.81) conditions than photopic conditions, whereas mesopic and dark conditions did not differ (*t*(23) = 2.25, *p* = 0.102, *d* = 0.46). *13 Hz Stimulation*. Thresholds were lower for mesopic than both dark (*t*(23) = 7.58, *p* < 0.001, *d* = 1.55) and photopic (*t*(23) = 12.31, *p* < 0.001, *d* = 2.51) conditions, and lower for dark compared to photopic conditions (*t*(23) = 4.73, *p* < 0.001, *d* = 0.97). *16 Hz Stimulation.* Thresholds were lower for mesopic than both the photopic (*t*(23) = 9.64, *p* < 0.001, *d* = 1.97) and dark (*t*(23) = 10.27, *p* < 0.001, *d* = 2.10) conditions, whereas dark and photopic conditions did not differ (*t*(23) = 2.54, *p* = 0.055, *d* = 0.52). *18 Hz Stimulation.* Thresholds were lower for the mesopic than both photopic (*t*(23) = 7.98, *p* < 0.001, *d* = 1.63) and dark (*t*(23) = 11.65, *p* < 0.001, *d* = 2.38) conditions, and photopic less than the dark condition (*t*(23) = 4.12 *p* = 0.001, *d* = 0.84). *20 Hz Stimulation.* Thresholds were lower for the mesopic than both photopic (*t*(23) = 3.25, *p* = 0.009, *d* = 0.67) and dark (*t*(23) = 10.71, *p* < 0.001, *d* = 2.19) conditions, and photopic less than dark condition (*t*(23) = 7.29, *p* < 0.001, *d* = 1.49).

The lowest thresholds within each lighting condition (across all frequencies) differed as a function of Lighting Condition (main effect: *F*(2,46) = 34.84, *p* < 0.001, $\eta_{p}^{2}$ = 0.602), with lower thresholds found in the mesopic condition (at 16 Hz) relative to each of the dark (at 10 Hz; *F*(1, 23) = 54.88, *p* < 0.001, $\eta_{p}^{2}$ = 0.705) and light (at 20 Hz; *F*(1, 23) = 66.42, *p* < 0.001, $\eta_{p}^{2}$ = 0.743) conditions. No difference was found between the lowest light and dark condition thresholds (*p* = 0.772).

For the dark condition, a linear model (AICc = 193.02) produced a better fit than the quadratic model (AICc = 194.34). For the mesopic condition, a quadratic model (AICc = 199.46) produced a better fit than the linear model (AICc = 289.71). For the photopic condition, a quadratic model (AICc = 37.02) produced a better fit than the linear model (AICc = 64.34).

The regression analyses (predicting threshold as a function of frequency) resulted in the following equations (see Fig. 2b):

**Dark**: Threshold = − 3.130 + 0.203 × Frequency; adj-R^2^ = 0.650, *p* < 0.001.
**Mesopic**: Threshold = 13.682 − 1.800 × Frequency + 0.056 × Frequency^2^; adj-R^2^ = 0.634, *p* < 0.001.
**Photopic**: Threshold = 6.587 − 0.655 × Frequency + 0.014 × Frequency^2^; adj-R^2^ = 0.905, *p* < 0.001.

Participants consistently reported that phosphenes in the mesopic and dark conditions increased in luminosity (compared to their background) as the stimulation intensity increased. Under photopic conditions, participants consistently reported that the flashing of the phosphene appeared to make their field of view seem darker compared to pre-stimulation perceived luminance levels, where the flashing alternated between a visible flash and the previous level of general luminosity. This distinction became stronger as stimulation intensity increased. Four participants reported seeing coloured phosphenes, however these reports were not consistent across those individuals, nor within individuals across sessions.

## Discussion

The aim of the study was to determine the relations between phosphene detection thresholds and both ambient lighting and tES stimulation frequency. Those relations enabled us to test whether: (1) the recently reported evidence of greater sensitivity to tES-induced phosphenes at 16 Hz^18,19,35^, as opposed to the standard view that sensitivity is greatest at 20 Hz^5,7,11,20^, and (2) whether this apparent contradiction in the literature was due to ambient lighting.

As can be seen in Fig. 2, each of the ambient lighting conditions had a unique phosphene threshold relation with stimulation-frequency, whereby thresholds increased with frequency in the dark condition, reduced with frequency in the photopic condition, and reduced with frequency from 10 to 16 Hz and increased from 16 to 20 Hz in the mesopic condition. Corresponding to this, threshold minima under dark, mesopic and photopic conditions were found at 10 Hz, 16 Hz and 20 Hz respectively. This demonstrates that both ambient lighting condition and stimulation frequency are important for determining the minimum current required to induce phosphenes. Correlation analyses showed consistent phosphene thresholds across sessions, indicating that order effects and the choice of which electrode was the cathode or anode had no significant effect on the results.

The lowest thresholds overall were found under mesopic conditions (at 16 Hz), with thresholds significantly higher under both dark (at 10 Hz) and photopic (at 20 Hz) conditions. This means that threshold estimates obtained using the standard photopic or dark conditions, regardless of frequency, will overestimate the current required to induce phosphenes. It follows that guidelines using phosphene detection to set exposure restrictions based on data obtained in dark or photopic conditions (e.g.^14^), may underestimate the effect of electric current on neural processes (by 56–60%). It is important to note that even though such exposure guidelines typically rely on research using magnetic fields (rather than tES) to induce phosphenes, in both cases the cause of the phosphene is the current flowing through neural tissue^11^, which stimulates the same physiological processes. It follows that the present results are also applicable to research using magnetic fields to induce phosphenes, and thus to low frequency electromagnetic field exposure guidelines.

The present data also resolve the apparent discrepancy between recent research, which found thresholds for phosphene detection at 16 Hz^18,19,35^, and studies reporting thresholds at either 10 or 20 Hz (e.g.^15,20^). That is, the present findings demonstrate that mesopic conditions result in greatest sensitivity at 16 Hz^18,19^, whereas dark and photopic conditions (similar to those in past studies) result in greatest sensitivity at 10 and 20 Hz tES respectively. There is thus no inconsistency, only predictable differences due to the differing ambient lighting conditions used. Overall, the results of this study relating to ambient lighting and frequency dependence are consistent across multiple forms of tES, whether using tACS^11,15,18^ or sinusoidal tDCS^19^. While there are differences in the levels of current required to induce phosphenes across studies, this is a likely product of different methodological choices, as there are a multitude of variables that can change this threshold. For example, even when using the same montage on the same sample, the threshold values can vary based on the size, shape and surface area of the electrodes, the material which the electrode is made from, and the conductivity medium selected (e.g., conductive gel, electrolyte-soaked sponges). Changing any of these variables will change the volume conduction characteristics of the overall circuit, resulting in different levels of current density at the retina^30^. As a result, while the comparison of thresholds across studies is of little value, the findings relating to frequency and lighting remain consistent despite any variations in stimulation methodology.

Although it is tempting to suggest that different physiological processes are being engaged during tES in the mesopic relative to dark and photopic conditions, a simpler explanation may be sufficient to explain the results. As can be seen in Fig. 2, the shape of the estimated distribution of thresholds in the mesopic condition matched that of the dark condition at frequencies below the approximate crossover point at 16 Hz, and also matched that of the photopic condition at frequencies above the 16 Hz crossover point. Taken together, it would thus appear that the mesopic condition may simply represent the combination of physiological processes normally engaged in each of the dark and photopic conditions.

Consistent with this hypothesis, there is evidence that the observed frequency dependence in tES-induced phosphene research can be explained by differences in the relative activity of rod-based and cone-based vision^36,37^. Cells related to rod vision, which are primed to respond in dark conditions, are maximally sensitive to stimulation at circa 10 Hz^38–40^, whereas cells related to cone vision, which are primed to respond in photopic conditions, are maximally sensitive to stimulation at circa 20 Hz^27,37^. In itself this would not explain the magnitude of threshold reduction in the mesopic condition (60% and 56%, relative to the dark and photopic conditions respectively), particularly given that 16 Hz is far from the ideal stimulation frequency for either rod- or cone-related cells. However, when coupled with what is known about the rod-cone processing delay, this would appear a viable hypothesis. That is, there is a delay between rod- and cone-related cell processing under mesopic conditions^41^, but as the stimulation frequency reaches approximately 15 Hz, rods and cones start to fire in phase, which increases the signal at both rods and cones and enhances the perceptibility of the stimulation^42^. This critical 15 Hz frequency also approximates the crossover point of the dark and photopic regression estimates (see Fig. 2b), indicating that the rod-cone phase delay mechanism may be behind the lower overall thresholds at the nearby 16 Hz frequency in mesopic conditions. Further research would be required to test this hypothesis.

While high levels of current can result in discomfort or pain at the site of stimulation^43^, these effects are typically found at stimulation strengths exceeding the maximum used in this study. The maximum strength of stimulation (1500 μA) in the present study was selected in order to avoid such side effects. One subject reported an unpleasant itching-like sensation at stimulation levels above 900 μA, however the sensation immediately ceased upon termination of the stimulation. Despite multiple enquiries during each testing session, no other participant reported any negative side effects, either during or after stimulation. Indeed, as the study deliberately kept current levels low to identify thresholds, this reduced the opportunity to obtain meaningful information about the phosphene experience more generally, which may otherwise have helped shed light on the underlying physiology responsible for phosphene induction. Of particular relevance is the degree to which phosphenes were perceived in chromatic (as opposed to achromatic) colour, as that could provide evidence for the relative mechanistic roles of rods and cones, as a function of frequency and lighting condition. However, given the low current strengths used in the study, only four participants reported seeing chromatic colour, and reports were not consistent across those individuals, nor within each individual across sessions. We thus do not believe that these anecdotal reports are sufficient to enable interpretation.

## Conclusion

The present study has shown that the apparent contradiction in the literature, in terms of tES stimulation frequency and phosphene detection threshold, was due to the different ambient lighting conditions used across past studies. That is, whereas thresholds under dark and photopic conditions are lowest for 10 and 20 Hz stimulation respectively, they do not represent overall thresholds, which occur at 16 Hz in mesopic conditions. The magnitude of threshold overestimation was very large (60 and 56% for dark and photopic conditions respectively), and thus important for application of tES research. Physiological considerations suggest that the lower thresholds in mesopic conditions, and particularly at 16 Hz stimulation, may be due to the involvement of both rod and cone photoreceptors, but further research is required to determine this. Importantly, our research also shows (for the first time) that dark, mesopic and photopic lighting conditions each have their own unique phosphene threshold relation with stimulation-frequency.

## Supplementary Information

Supplementary Table 1.

## Data Availability

The dataset resulting from this experiment is available from the corresponding author upon reasonable request.
